# Up-regulation of brain-derived neurotrophic factor in primary afferent pathway regulates colon-to-bladder cross-sensitization in rat

**DOI:** 10.1186/1742-2094-9-30

**Published:** 2012-02-15

**Authors:** Chun-Mei Xia, Melisa A Gulick, Sharon J Yu, John R Grider, Karnam S Murthy, John F Kuemmerle, Hamid I Akbarali, Li-Ya Qiao

**Affiliations:** 1 Departments of Physiology and Biophysics, Virginia Commonwealth University School of Medicine, 1220 East Broad Street, PO Box 0551, MMRB 5038, VA 23219 Richmond, Virginia; 2 Department of Internal Medicine, Virginia Commonwealth University School of Medicine, Richmond, Virginia; 3 Department of Pharmacology and Toxicology, Virginia Commonwealth University School of Medicine, Richmond, Virginia

**Keywords:** BDNF, Afferents, Cross-sensitization, Colon, Bladder

## Abstract

**Background:**

In humans, inflammation of either the urinary bladder or the distal colon often results in sensory cross-sensitization between these organs. Limited information is known about the mechanisms underlying this clinical syndrome. Studies with animal models have demonstrated that activation of primary afferent pathways may have a role in mediating viscero-visceral cross-organ sensitization.

**Methods:**

Colonic inflammation was induced by a single dose of tri-nitrobenzene sulfonic acid (TNBS) instilled intracolonically. The histology of the colon and the urinary bladder was examined by hematoxylin and eosin (H&E) stain. The protein expression of transient receptor potential (TRP) ion channel of the vanilloid type 1 (TRPV1) and brain-derived neurotrophic factor (BDNF) were examined by immunohistochemistry and/or western blot. The inter-micturition intervals and the quantity of urine voided were obtained from analysis of cystometrograms.

**Results:**

At 3 days post TNBS treatment, the protein level of TRPV1 was increased by 2-fold (*p *< 0.05) in the inflamed distal colon when examined with western blot. TRPV1 was mainly expressed in the axonal terminals in submucosal area of the distal colon, and was co-localized with the neural marker PGP9.5. In sensory neurons in the dorsal root ganglia (DRG), BDNF expression was augmented by colonic inflammation examined in the L1 DRG, and was expressed in TRPV1 positive neurons. The elevated level of BDNF in L1 DRG by colonic inflammation was blunted by prolonged pre-treatment of the animals with the neurotoxin resiniferatoxin (RTX). Colonic inflammation did not alter either the morphology of the urinary bladder or the expression level of TRPV1 in this viscus. However, colonic inflammation decreased the inter-micturition intervals and decreased the quantities of urine voided. The increased bladder activity by colonic inflammation was attenuated by prolonged intraluminal treatment with RTX or treatment with intrathecal BDNF neutralizing antibody.

**Conclusion:**

Acute colonic inflammation increases bladder activity without affecting bladder morphology. Primary afferent-mediated BDNF up-regulation in the sensory neurons regulates, at least in part, the bladder activity during colonic inflammation.

## Background

Clinical evidence has shown sensory cross-sensitization between the urinary bladder and the distal colon [1-5]. Patients with inflammatory bowel disease (IBD) are more likely to experience nocturia and some forms of urinary urge incontinence compared to the non-IBD population [3]. Other forms of cross-organ sensitization are also observed in experimental animals. Rats or mice induced for cystitis or colitis exhibit decreases in the threshold to stimulation of the hindpaw demonstrating a viscero-somatic cross-sensitization [6-8]. Inflammation in the lower extremities also causes an enhanced response to colorectal distension [6,9,10], suggesting that visceral sensitivity can also be influenced by the irritation of non-visceral organs. Recent studies in anesthetized animals have shown that colonic irritation leads to neurogenic cystitis as manifested by irritative micturition patterns and increases in micturition frequency [11,12]. Conversely cystitis induced by cyclophosphamide also increases colorectal afferent sensitivity in mice [13]. These observations indicate a broad phenomenon between organ to organ sensory cross-interaction.

The currently proposed mechanism and pathways underlying cross-organ sensitization may involve activation of primary afferent pathways [14-16]. Rodents with experimentally induced colonic inflammation exhibit an enhanced firing of bladder C-fibers in response to bladder distension [12,17]. Activation of primary afferent pathways by one form of peripheral organ irritation may lead to cross-activation of the primary afferent neurons projecting to another peripheral organ [18,19] or lead to central sensitization in the spinal cord [20]. This is particularly true with colonic inflammation which not only sensitizes colonic afferent neurons but also alters the molecular profiles of bladder afferent neurons in the dorsal root ganglia (DRG). It has been reported that colonic inflammation significantly increases the expression level of calcitonin gene-related peptide (CGRP) [19], and increases the currents of TTX-resistant (TTX-R) Na^+ ^channels [18] in specifically labeled bladder sensory neurons.

The transient receptor potential (TRP) ion channel of the vanilloid type 1 (TRPV1) is involved in many systems during inflammation and sensory sensitization [21-26]. TRPV1 receptor antagonist has prevented the visceral hypersensitivity to intracolonic mechanical and chemical stimulation [24]. Animals deficient in TRPV1 exhibit reduced responses of primary sensory afferent fibers to mechanical distension of the colon [22]. The activity of TRPV1 is regulated by potent agonist such as resiniferatoxin (RTX) in a biphasic fashion. Acute RTX treatment results in enhanced TRPV1 activity, while a prolonged treatment with RTX produces desensitization of the receptor [27-31]. In animal studies, a prolonged RTX treatment is demonstrated for its effects on desensitization of unmyelinated nociceptive C-fiber afferents [27,29,30]; in humans, RTX has also been suggested for therapeutic intervention of visceral disorders [28,32,33]. In our previous studies, we have successfully used RTX to block colonic inflammation-induced TrkA up-regulation in the colonic afferent neurons [34]. These studies suggest a role of TRPV1 in inflammation-induced sensory plasticity.

The brain-derived neurotrophic factor (BDNF) has been postulated to play an important role in inflammation-induced sensory hypersensitivity by modulating the sensitivity of primary afferents [19,35-37]. Blockade of BDNF action with spinal intrathecal injection of BDNF/TrkB antiserum has significantly reduced inflammation-induced hyperalgesia [38,39]. Systemic knockdown of BDNF activity by intraperitoneal injection of BDNF neutralizing antibody also reverses colitis-induced colonic hypersensitivity [40]. Conversely, intraperitoneal injection of BDNF protein significantly enhances colonic sensitivity and decreases the colonic reaction threshold in healthy rats [40]. A recent study has shown that with colitis BDNF(+/-) mice exhibit weaker visceral responses to colorectal distension and lower sensitivity in the colon than BDNF(+/+) mice [41]. These findings imply that the increased level of BDNF in primary sensory neurons in the DRG during colonic inflammation [37] may have a role in mediating visceral hypersensitivity.

## Materials and methods

### Intracolonic instillation of TNBS to induce localized colonic inflammation

Adult male Sprague-Dawley rats (150-200 g) from Harlan Sprague Dawley, Inc. were used for this study. To induce inflammation in the distal colon, fasted rats were anesthetized (2% isoflurane, SurgiVet, Smiths Medical PM, Inc. Waukesha, WI). A single dose of TNBS was instilled into the lumen of the colon 6 cm proximal to the anus at a dose of 90 mg/kg (1.5 mL/kg of 60 mg/mL solution in 50% EtOH) through a polyethylene (PE) catheter. Control animals received a similar volume of 50% EtOH. Examinations were performed 3 days post TNBS or vehicle treatment.

### Harvest of tissues

The distal colon and the urinary bladder from control and the inflamed animals were removed, fixed with 4% paraformaldehyde and sectioned transversely at a thickness of 10 μm, or the distal colon underwent whole mount dissection for separation of the mucosal, submucosal, and muscle layers. The DRGs were sectioned at a thickness of 20 μm. Tissues from control and experimental animals were handled in an identical manner.

### Hematoxylin and eosin (H &E) stain to assess the morphology and inflammation of the colon and the urinary bladder

Transverse sections of the distal colons and urinary bladders from all animals were stained with an H & E staining kit according to the protocol provided by the manufacture (Richard-Allan-Scientific, Kalamazoo, MI). The sections were examined with a Zeiss brightfield microscope. The histology score was graded to reflect the severity of the visceral inflammation (1, no inflammation; 2, very low inflammation; 3, low level of infiltration; 4, high level of infiltration and vascular density; 5, transmural infiltrations and high vascular density). The increases in the thickness of the muscular wall, the width of the submucosal space (lamina propria), and the depth of the mucosal layer were also considered as signs of inflammation.

### Immunohistochemistry

Sections and the tissue layers were incubated with blocking solution containing 3% normal donkey serum in PBST (0.3% Triton X-100 in 0.1 M PBS, pH 7.4) for 30 min followed by rabbit anti-TRPV1 (1:1000, Chemicon International Inc., CA), rabbit anti-BDNF (1:500, Santa Cruz, CA), and/or sheep anti-BDNF (1:200, Chemicon International Inc., CA) primary antibodies overnight at 4°C. After rinsing (3 × 10 min with 0.1 M PBS), tissues were incubated with Alexa 594-conjugated species-specific secondary antibody for 2 hours at room temperature. Some of the tissues were also co-stained with guinea pig anti-PGP9.5 primary antibody (1:1000, Chemicon International Inc., CA) followed by Alexa 488-conjugated species-specific secondary antibody. Following washing, the slides were coverslipped with Citifluor (Citifluor Ltd., London). Immunostaining in the absence of primary or secondary antibody was assessed for background evaluation. The specificity of the antibodies was also evaluated with western blot or pre-absorption assay.

Tissue sections were viewed and analyzed under a Zeiss fluorescent photomicroscope. DRG neurons expressing BDNF were counted in 4 to 6 sections of each DRG, and expressed as mean ± SE for n animals. Similar sized sections were chosen using the microscope measurement program and all the positive cells were counted in the sections. The results were expressed as number of cells per section. To avoid double counting, we have chosen every third DRG section for BDNF immunostaining. For analysis of the TRPV1 immunoreactivity in the distal colon, three random microscopy fields were chosen from each section with caution to avoid field overlap. Each field was positioned to view a similar morphological area covering the entire width of the colon that showed positive TRPV1 stain. The number of the positive stain in each field was counted and averaged within each animal. The size of the field was measured. The results were expressed as number of TRPV1 stain per mm^2^.

### Protein extraction and western blot

Segments from the distal colon were homogenized in solubilization buffer containing 50 mM Tris-HCl, 150 mM NaCl, 1 mM EDTA, 1% Triton X-100, and 100 mM NaF supplemented with protease and phosphatase inhibitor cocktails (Sigma, St. Louis, MO). The homogenate was centrifuged at 20,200 g for 10 min at 4°C, and the supernatant was removed to a fresh tube for further analysis. The protein concentration was determined using Bio-Rad DC protein assay kit.

Proteins were separated on a 10% SDS-PAGE gel and transferred to a nitrocellulose membrane. The membrane was blocked with 5% milk in Tris-buffered saline for 1 hour and then incubated with rabbit ant-TRPV1 (1:1000) antibody followed by goat anti-rabbit IRDye 800 CW. For internal loading control, the same membrane was stripped and re-probed with antibody against β-actin (1:2000, Sigma). The bands were identified and analyzed with the ODYSSEY infrared imaging system (Li-cor Biosciences, Lincoln, NE). The expression level of the target protein in control animal from each independent experiment was considered as 1, and the relative expression level of the target protein in experimental animals was adjusted as a ratio to its internal loading control in each independent experiment.

### Drug delivery to animals

To specifically desensitize or destroy the colonic afferents, RTX was dissolved in 10% Tween-80, 10% ethanol, and normal saline and administered intracolonically (into the lumen of the colon) at a dose of 250 μg/kg/2 mL 10 days prior to test. BDNF antibody or control IgG (100 μg/kg body weight, Santa Cruz, CA) was delivered into the subarachnoid space of the spinal cord via an intrathecal (i.t.) catheter (No. 0007740, Alzet) connected to an osmotic-pump (Alzet 2001) four days before TNBS instillation, and was continuously delivered until the day of examination which was 3 days post TNBS treatment. The tip of the catheter was positioned at the L4-L5 spinal level and was confirmed after euthanasia of the animals. This delivery site was chosen because of its position located in the middle of L1 and S1 segments where we found that BDNF was up-regulated during colitis. This procedure was done under anesthesia (2.5% isoflurane).

### Measurement of urinary bladder micturition pattern

Under anesthesia (2.5% isoflurane), a lower midline abdominal incision was performed and a PE-50 catheter was inserted into the urinary bladder through a small incision made by an 18 G needle at the tip of the bladder dome. The catheter was secured in place with a 6-0 nylon purse-string suture. The distal end of the tubing was externalized at the back of the neck and the rat was allowed to recover. Four days after the catheter was implanted, the animal was placed into a recording chamber (Med Associates, St. Albans, VT). The distal end of the catheter was connected *via *a T tube to a pressure transducer and a syringe pump (Med Associates, St. Albans, VT). Animals were allowed to recover from anesthesia and explore the new environment. The baseline of the bladder pressure was obtained before a 0.9% saline solution was infused into the bladder at a rate of 9 mL/h according to the manufacture's suggestions. After initial stabilization, the micturition pattern of the urinary bladder was recorded for a period of 20 to 30 min. No particular time of the day was chosen for each animal. Control animals and animals receiving treatment were randomly assigned for testing during the day. The bladder inter-micturition intervals (seconds between two voiding) were measured, analyzed and expressed as seconds/interval. The quantity of urine of each voiding was automatically weighed by a scale integrated in the system, and was analyzed and expressed as gram (g).

### Statistical analysis

The results from each study were presented as mean ± SE for *n *animals. Comparison between control and experimental groups was made by using a one-way ANOVA. When two groups were compared, a student's *t *test was used. Differences between means at a level of *p *≤ 0.05 were considered to be significant.

## Results

### Colonic inflammation increased TRPV1 expression in the distal colon but not in the urinary bladder

**Figure 1:**
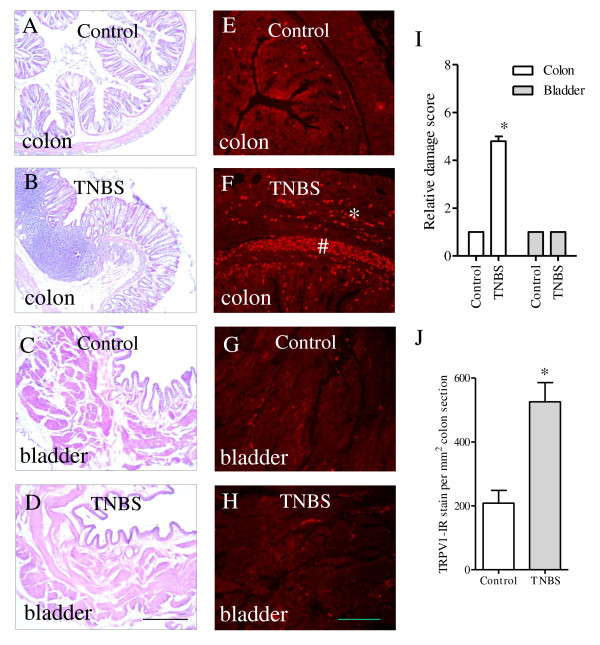
**Histologic changes and TRPV1 immunoreactivity in the distal colon and the urinary bladder during colonic inflammation**. TNBS treatment resulted in inflammatory response of the distal colon (A-B) and increased immunoreactivity of TRPV1 in the distal colon (E-F). Colonic inflammation was not accompanied by bladder inflammation (C-D), or changes in the TRPV1 immunoreactivity in the urinary bladder (G-H). Four independent experiments showed consistent results. Quantification of the results were shown in I and J. Bar = 300 μm in A-D; Bar = 50 μm in E-H. *, *p *< 0.05.

**Figure 2:**
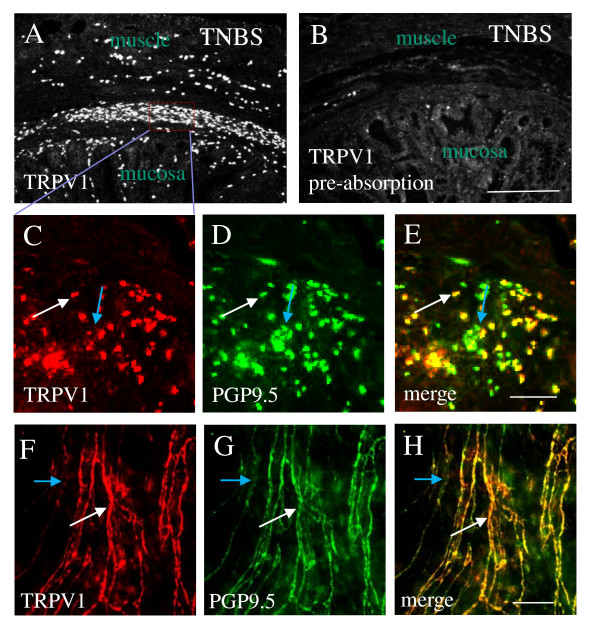
**TRPV1 immunoreactivity in PGP9.5-positive structures in the distal colon**. The specificity of the TRPV1 antibody was examined by pre-absorption assay showing that pre-incubation of TRPV1 antibody with a TRPV1 control peptide completely abolished the TRPV1-like immunoreactivity detected in the inflamed distal colon (compare B to A). Double immunostaining of the transverse section of the inflamed distal colon showed that TNBS-increased TRPV1 protein expression was localized in PGP9.5 positive structures (C-D, white arrows). Double immunostaining of the whole mount preparation of the submucosal plexus showed that TRPV1 was expressed in PGP9.5 positive fibers (F to H, white arrows). Note that not all PGP9.5 positive structures contain TRPV1 (C-H, blue arrows). Bar = 50 μm.

We compared the histology of the distal colon and the urinary bladder before and after induction of colonic inflammation. H&E staining (Figure 1A-D) showed that the distal colon had the appearance of severe inflammatory infiltration, edema, loss of the mucosal architecture, high level of vascular density, and increases in the thickness of muscular wall and the width of the submucosal spaces (compare Figure 1B to 1A). The histology of the urinary bladder appeared normal in terms of the structure and folds of the urothelium, the size of the gap of the suburothelium space, and the thickness of the muscular wall (compare Figure 1D to 1C; Figure 1I shows the relative damage scores of the colon and the urinary bladder). To characterize the sensory profile in the distal colon and the urinary bladder, we examined the expression of the sensory marker TRPV1 in these organs with immunohistochemistry and western blot techniques. During colonic inflammation, the TRPV1-like immunoreactivity was dramatically enhanced within the muscular layer (indicated by *) and the submucosal plexus (indicated by #) of the distal colon (Figure 1F) when compared to the non-inflamed distal colon (Figure 1E, J). The density of TRPV1 immunoreactivity was not altered in the urinary bladder before (Figure 1G) and after (Figure 1H) induction of colonic inflammation. To examine the specificity of the TRPV1-immunoreactive structures in the distal colon, we performed pre-absorption assay with a TRPV1 blocking peptide (5 μg/mL, Santa Cruz, CA) and found that this blocking peptide completely abolished the TRPV1-like immunoreactivity in both regions of the muscular layer and the submucosal area (compare Figure 2B to 2A), suggesting that the punctuated staining in the distal colon by this TRPV1 antibody was specific TRPV1 immunoreactivity. Further examination with double immunostaining showed that the TRPV1 immunoreactivity in the inflamed distal colon was expressed in PGP9.5 positive structures (Figure 2C-H). These co-localization studies were conducted with transverse sections (Figure 2C-E) and the whole-mount preparation of the inflamed distal colon (Figure 2F-H). Both methods demonstrated consistent results showing that all TRPV1 immunoreactivity was expressed in PGP9.5 positive structures (Figure 2C-H, white arrows), but not all PGP9.5 positive structures contained TRPV1 (Figure 2C-H, blue arrows).

**Figure 3:**
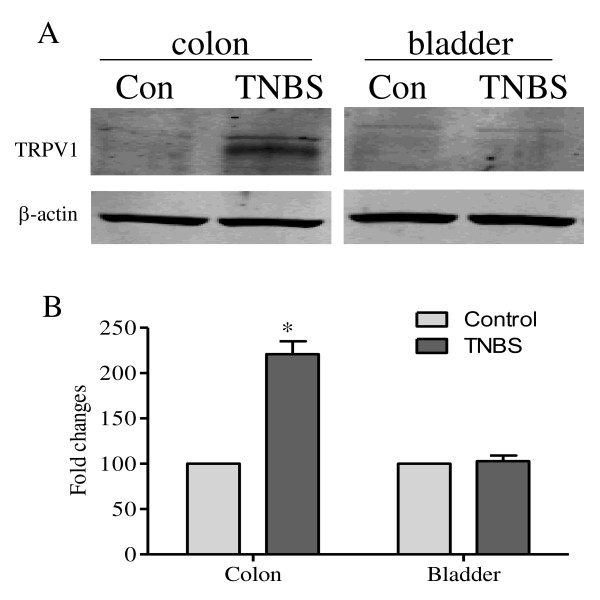
**Western blot of TRPV1 in the distal colon and the urinary bladder**. Western blot results showed that TNBS treatment increased the protein level of TRPV1 in the distal colon by 2-fold (A and B, colon). The level of TRPV1 was not changed in the urinary bladder post TNBS treatment (A and B, bladder). *, *p *< 0.05. n = 5 for each experimental group.

Western blot results confirmed the findings above showing that the protein expression level of TRPV1 was increased by 2-fold (*p *< 0.05) at day 3 post induction of colonic inflammation (Figure 3). In contrast, colonic inflammation did not alter the expression level of TRPV1 in the urinary bladder (Figure 3).

### Colonic inflammation-induced BDNF expression in the dorsal root ganglia was attenuated by prolonged pre-treatment with RTX

**Figure 4:**
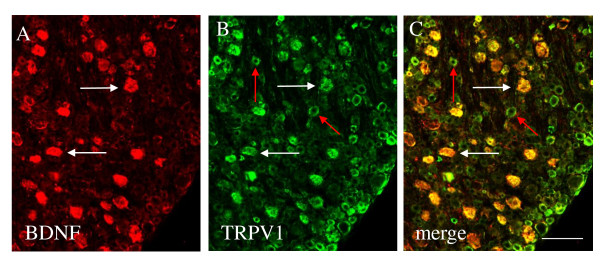
**BDNF expression in TRPV1 neurons in L1 DRG**. Virtually all BDNF immunoreactivity (A, red cells) was expressed in TRPV1 neurons (B, green cells) in the DRG during colonic inflammation. However, a subpopulation of TRPV1 positive neurons (C, green cells, red arrows) did not express BDNF. Bar = 60 μm.

Our previous studies (37) showed that the BDNF mRNA level was increased in the L1 and S1 DRG at 3 days of colitis. However BDNF protein level was only increased in the L1 DRG but not in the S1 DRG at this time point. Conversely, BDNF protein was increased in the S1 spinal cord due to BDNF release from the S1 DRG (37). To examine the phenotype of BDNF immunoreactive neurons in the DRG in order to validate a role of BDNF in sensory plasticity, we performed double immunostaining of BDNF and the nociceptive marker TRPV1 in the L1 DRG at 3 days of colitis. Results showed that BDNF immunoreactivity was expressed in nociceptors identified by TRPV1 immunostaining (Figure 4C, yellow cells, white arrows). Virtually all BDNF was expressed in TRPV1 neurons; however, a subpopulation of TRPV1 immunoreactive neurons (Figure 4C, green cells, red arrows) did not express BDNF.

**Figure 5:**
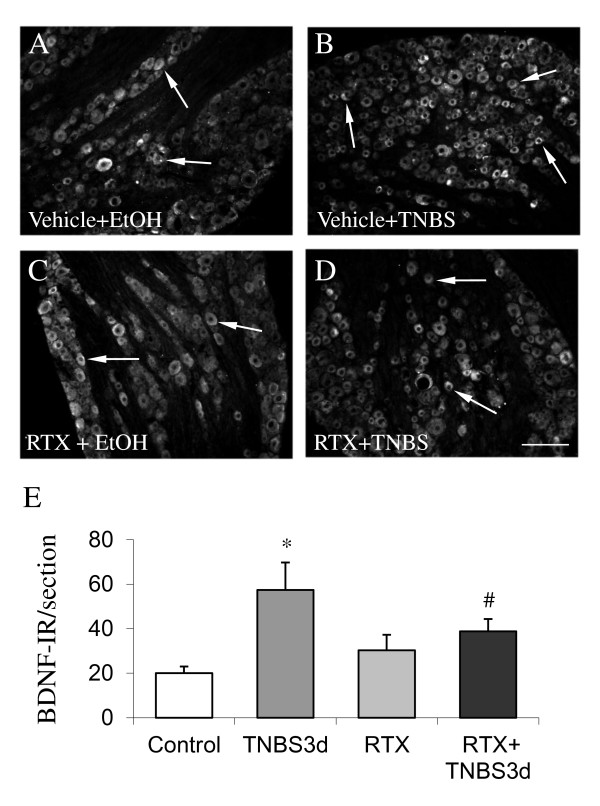
**Prolonged pre-treatment with RTX blocked BDNF expression in L1 DRG during colonic inflammation**. In vehicle-treated animals, colonic inflammation increased the level of BDNF in the L1 DRG at 3 days post TNBS treatment (A, B, E). Following prolonged RTX pre-treatment, the level of BDNF in L1 DRG from the inflamed animals was attenuated to almost normal level (D, E). Bar = 40 μm, *, *p *< 0.05 vs. control; #, *p *< 0.05 vs. TNBS-treated. n = 4 animals for each experimental group.

To examine if the activation of TRPV1 primary afferents (presumably C-fibers) had a role in BDNF up-regulation in sensory neurons during colonic inflammation, we determined the level of BDNF in animals treated with vehicle (10% Tween-80, 10% ethanol, and normal saline) + 50% EtOH (Figure 5A), vehicle + TNBS (Figure 5B), RTX + EtOH (Figure 5C), or RTX + TNBS (Figure 5D). Animals were pre-treated with either vehicle or RTX for one week and then were treated with either EtOH or TNBS for 3 days. The BDNF expression was examined by immunohistochemistry. Results showed that prolonged pre-treatment with RTX significantly attenuated colonic inflammation-induced BDNF up-regulation examined in L1 DRG (Figure 5E). RTX had no effects on the expression level of BDNF in DRG from non-inflamed animals (Figure 5E).

### Blockade of BDNF attenuated colonic inflammation-induced bladder overactivity

**Figure 6:**
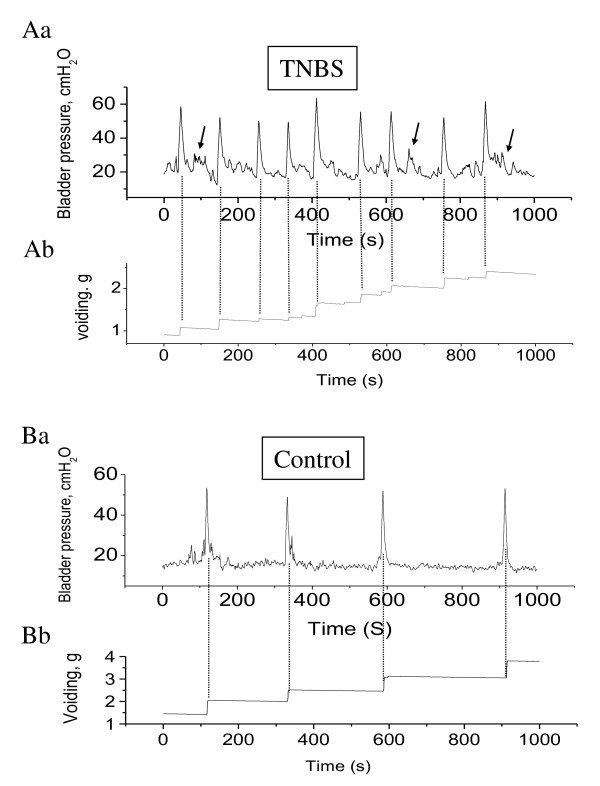
**Cystometrograms in control animals and animals with colonic inflammation**. The bladder inter-micturiton intervals (Aa and Ba) and the quantity of solution voided (Ab and Bb) were recorded in unrestrained conscious animals. The dotted vertical lines align the time points for urination, thus the distance between the dotted lines (or the peak of the micturition pressure) represents the inter-micturition intervals. Arrows indicate the non-voiding contractions (increases in the intravesicle pressure (Aa) but no voiding collected (Ab) corresponding to these urges).

**Figure 7:**
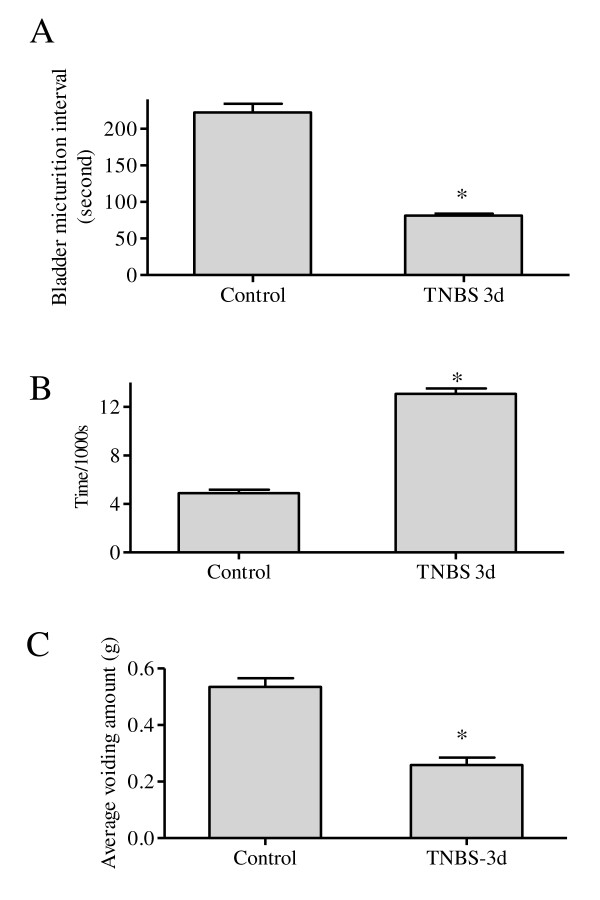
**Colonic inflammation increased the activity of the urinary bladder**. Colonic inflammation decreased the micturition intervals (A) and increased the micturition frequency (B). Colonic inflammation also decreased the average amount of solution per voiding (C). *, *p *< 0.05 vs. control. n = 4 animals for each treatment.

In conscious animals, colonic inflammation significantly decreased the micturition intervals when compared to control (control: 220.6 ± 12.1 seconds; TNBS: 81.2 6 ± 2.8 seconds; *p *< 0.01; Figure 6 and 7A). During colonic inflammation, animals also exhibited non-voiding contractions (arrows in Figure 6Aa), which were not seen in control animals (Figure 6Ba). To confirm the results, the micturition frequency was independently counted and calculated as 4.9 ± 0.3 times within a 1000-second time period prior to TNBS treatment. The micturition frequency was increased by 2.5-fold at day 3 post induction of colonic inflammation (Figure 7B). During micturition, the quantity of urine voided was weighed by a scale included in the system, and was automatically recorded by the program (Figure 6Ab, Bb). TNBS treatment significantly reduced the amount of each voiding (Figure 7C).

**Figure 8:**
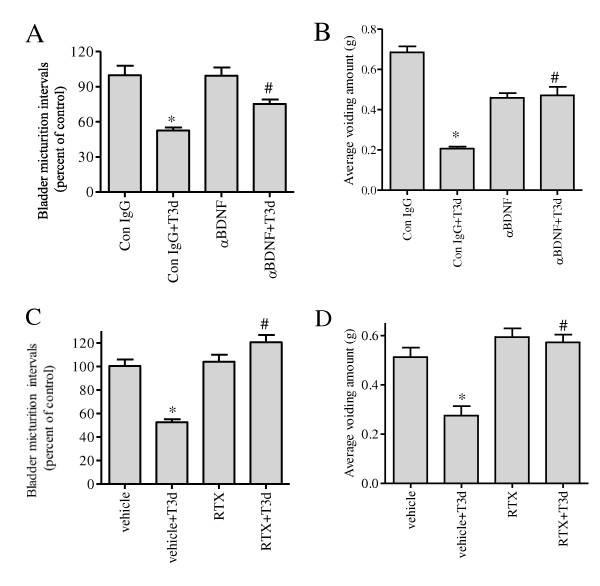
**Effects of BDNF antibody and RTX on the urinary bladder activity during colonic inflammation**. Spinal intrathecal BDNF neutralizing antibody reversed the colonic inflammation-induced decreases in micturition intervals by 75% (A), and also reversed the decreased voiding amount measured by a scale (B). Pre-treatment with RTX reversed the decreased micturition intervals to normal level (C), and also reversed the decreases in voiding quantity caused by colonic inflammation. n = 3 to 5 animals for each treatment. *, *p *< 0.05 vs vehicle or control IgG; #, *p *< 0.05 vs TNBS + vehicle or TNBS + IgG.

The role of BDNF in mediating bladder function was examined by intrathecal infusion of BDNF neutralizing antibody to the spinal cord with an osmotic-pump. Results showed that BDNF neutralizing antibody partially reversed the decreases in micturition intervals caused by colonic inflammation (Figure 8A). BDNF antibody treatment also reversed the reduction in the quantity of urine per voiding during colonic inflammation (Figure 8B). Since the up-regulation of BDNF in the DRG of the inflamed animals was blocked by pre-treatment with RTX (Figure 5), we examined the effects of RTX pre-treatment on bladder activity during colonic inflammation. When the animals were pretreated with intracolonic instillation of RTX one week prior to induction of colonic inflammation, the decreases in micturition intervals were reversed to normal level (Figure 8C). The antagonism of RTX also reversed the reduction in the quantity of urine per voiding during colonic inflammation (Figure 8D).

## Discussion

The present study demonstrates that colonic inflammation increases the expression level of TRPV1 in the distal colon but not in the urinary bladder. Colonic inflammation-induced BDNF is expressed in TRPV1 nociceptive neurons and is attenuated by blockade of primary afferent activity with prolonged pre-treatment with RTX. Neutralization of BDNF in the DRG/spinal cord with intrathecal infusion of a BDNF antibody reverses bladder overactivity during colonic inflammation. These results suggest a role of primary afferent -mediated BDNF up-regulation in viscero-visceral cross-organ sensitization.

BDNF is a member of the neurotrophin family of growth factors consisting of nerve growth factor (NGF), neurotrophin-3 (NT-3) and NT-4. It has long been implied that BDNF plays a significant role in neuronal plasticity especially the long-term potentiation (LTP) of the central nervous system [42]. Recent studies have also suggested a role of BDNF in modulating sensory activity in the peripheral nervous system. After peripheral inflammation, BDNF is synthesized in the primary sensory neurons in DRG where it facilitates intracellular signal transduction and gene expression at the dorsal horn of the spinal cord *via *anterograde transport [37,43,44]. As demonstrated in the present study, BDNF in the DRG is mainly expressed in TRPV1 nociceptive neurons. Blockade of primary afferents with the neurotoxin RTX reverses the BDNF up-regulation in the DRG during colonic inflammation, suggesting that signaling arising from the inflamed distal colon facilitates BDNF expression in the sensory neurons in DRG. Studies by us and others have demonstrated that colonic inflammation increases the level of NGF and/or neural activity in the inflamed colon [34,45,46]. NGF receptor TrkA is able to retrogradely transport from the inflamed colon to the DRG [34], where it may activate intracellular signaling molecules and regulate neuronal plasticity. The role of NGF/TrkA in regulating BDNF expression in the DRG has also been illustrated by previous studies showing that NGF treatment increases BDNF expression in the TrkA/CGRP peptidergic DRG neurons and almost 90% of TrkA DRG neurons express BDNF [47].

In addition to changes in the neurochemical coding of the sensory neurons during colonic inflammation, one of the major physiological alterations accompanying colonic inflammation is the bladder hyperactivity [7,11,12]. Analysis of cystometrograms reveals that the average inter-micturition intervals in control animals are 220 seconds when we infuse the urinary bladder with a saline solution at a speed of 9 mL/h. This paradigm results in, by calculation, an average of 0.55 mL infusing volume per micturition cycle, which is close to the directly measured amount of solution voided (an average of 0.537 g per micturition cycle). These results indicate that there was no leakage of solution into the abdomen from the intravesicle catheter insertion site during saline infusion. During colonic inflammation, we have found that the inter-micturition intervals are decreased and the quantity of urine voided is also decreased suggesting bladder overactivity in these animals. The bladder overactivity in colonic inflamed animals has also been confirmed *via *a non-invasive procedure in which the urine is collected naturally onto an underneath filter paper directly from the unrestrained nonoperated animals (Additional file 1). Analysis of the urine spots on the filter paper reveals that the animals excrete fewer times (i.e., lower number of urine drops) with larger volumes per drop (i.e., bigger urine spots) before induction of colonic inflammation. TNBS-treated animals void more frequently (i.e., higher number of urine drops) with smaller quantities of urine per voiding (i.e., smaller urine spots) (Additional file 1). Both techniques used in the current study demonstrate that colonic inflammation indeed induces bladder hyperactivity with minimum change in the morphology of the urinary organ, suggesting a neurogenic mechanism in colon-to-bladder cross-sensitization. It is noteworthy that there are increased numbers of non-voiding contractions detected in the inflamed animals. These non-voiding contractions may be due to the hyperactivity of the urinary bladder *per se*, or due to the abdominal contraction pressure transference to the viscera in the inflamed animal. Further examination of the activity of the abdominal wall will identify the mechanisms.

The mechanisms and pathways that mediate colon-to-bladder cross-organ sensitization have been vigorously studied in the past years. A growing body of evidence suggests that activation of the primary afferent pathway and a neural cross talk or interaction in the DRG and spinal cord has a significant role in mediating cross-organ sensitization [12,14-16,18,19]. The primary afferents that innervate the urinary bladder or the distal colon are located in the same spinal segments [19,48,49]. Thus, irritation of one visceral organ, such as the distal colon in the present study, may lead to activation of the primary afferent neurons projecting to this organ and cross-activation of the nearby afferent neurons projecting to a different viscus such as the urinary bladder. An experiment involving injection of a viral vector encoding NGF to the urinary bladder demonstrates that over-expression of NGF triggers the hypersensitivity of remote organs including the distal colon [6]. During colonic inflammation, the level of NGF is significantly increased in the distal colon [34,45]; the elevated NGF may have a role in triggering bladder hyperactivity by modulating the plasticity of sensory neurons. Modified bladder spinal reflex may regulate the detrusor muscle contractility and/or the activity of the urethral sphincter leading to increased voiding frequency.

BDNF elevated in the sensory neurons during colonic inflammation may modulate the bladder sensory activity. The level of BDNF high affinity receptor TrkB is increased in bladder afferent neurons during colonic inflammation [19]. The accumulation of TrkB in bladder afferent neurons may enhance the responsiveness of these neurons to BDNF, thus leading to changes in plasticity of these neurons. In DRG neuron culture, BDNF increases the expression level of CGRP [19], an excitatory neurotransmitter that is also up-regulated in the bladder afferent neurons during colonic inflammation [19], suggesting a possible role of BDNF in modulating bladder afferent excitability.

The role of BDNF in regulating visceral sensitivity has been suggested by a previous study showing that intraperitoneal injection of BDNF neutralizing antibody attenuates colonic inflammation-induced colonic hypersensitivity [40]. To specifically target the BDNF that is expressed in the DRG and spinal cord [37], in the present study a specific BDNF neutralizing antibody was infused into the DRG/spinal space under the dural membrane. This treatment regime also blocks colonic inflammation-induced bladder hyperactivity. BDNF that is up-regulated in the sensory neurons following colonic irritation may be released into the extracellular space within the DRG and the spinal cord. The action of BDNF is then blocked by the intrathecal BDNF antibody that further blocks the sensitivity of the bladder afferent neurons and modulates the urinary bladder function. Bladder afferent nerves consist of myelinated Aδ-fibers and unmyelinated C-fibers projecting to the lumbosacral spinal level which is also of major importance in controlling colonic function [50,51]. Activation of the C-fibers during colonic inflammation is apparent from our studies showing that the level of TRPV1 is significantly increased in nerve fibers in the inflamed distal colon. Blockade of the activity of these nerves with prolonged pre-treatment with RTX has also blocked colonic inflammation-induced bladder overactivity.

## Conclusion

The present study demonstrates that colonic inflammation causes neurogenic bladder overactivity. This cross-organ sensitization is regulated by primary afferent-mediated BDNF up-regulation in the DRG/spinal cord. The findings in the current study suggest a potential therapeutic role of BDNF analogs in the treatment of visceral hypersensitivity.

## Abbreviations

BDNF: Brain-derived neurotrophic factor; CGRP: Calcitonin gene-related peptide; DRG: Dorsal root ganglia; IBD: Inflammatory bowel disease; H&E: Hematoxylin and eosin; LTP: Long-term potentiation; NGF: Nerve growth factor; NT: Neurotrophin; PE: Polyethylene; RTX: Resiniferatoxin; TNBS: Tri-nitrobenzene sulfonic acid; TRPV1: Transient receptor potential ion channel of the vanilloid type 1.

## Grants

DK077917 to LYQ; DK034153 to JRG; DK028300 to KSM; DK049691 to JFK; DK046367 to HIA.

## Competing interests

The authors declare that they have no competing interests.

## Authors' contributions

CMX, MAG, LYQ designed and conducted most of the experiments. SJY, JRG designed and conducted some of the experiments CMX, LYQ wrote most of the manuscript. All authors analyzed the data, revised the manuscript and gave final approval for publication.

## Supplementary Material

Additional file 1 **The urine spots (circles) collected onto filter papers from control animals (A) and animals with 3 days of colitis (B)**. The Figure shows collections within a 30-min period. Bar = 2 cm. Click here for file
