# Supplementary figures and images for: Up-regulation of brain-derived neurotrophic factor in primary afferent pathway regulates colon-to-bladder cross-sensitization in rat
Source: J Neuroinflammation. 2012 Feb 15;9:30. doi: 10.1186/1742-2094-9-30 (PMC3298724; doi:10.1186/1742-2094-9-30)

## Supplemental Data

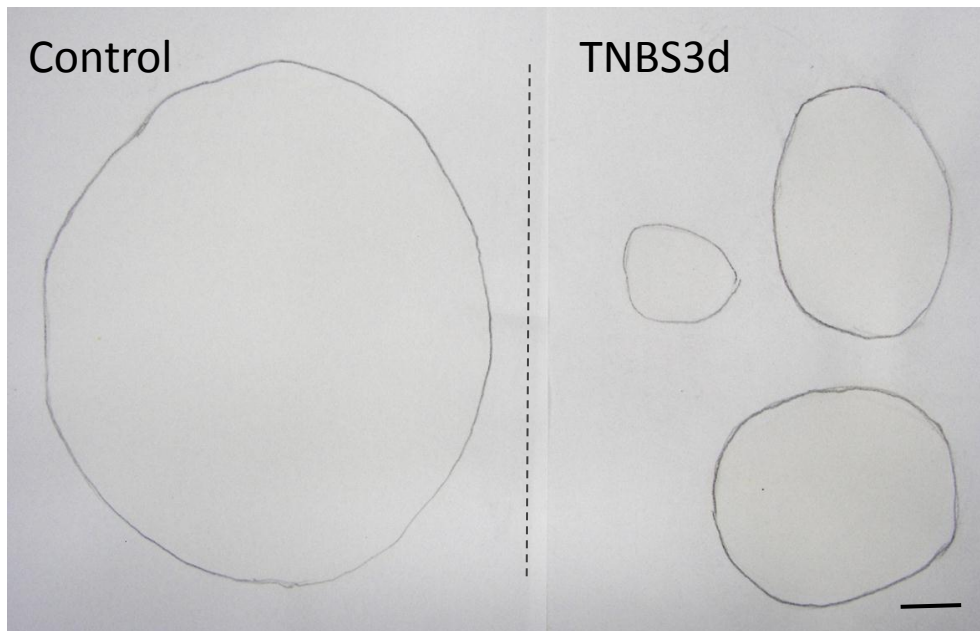

Supplement: Additional file 1 — The urine spots (circles) collected onto filter papers from control animals (A) and animals with 3 days of colitis (B). The Figure shows collections within a 30-min period. Bar = 2 cm. [file 1742-2094-9-30-S1.PDF]
